# A Large Scale Biorational Approach Using *Bacillus thuringiensis israeliensis* (Strain AM65-52) for Managing *Aedes aegypti* Populations to Prevent Dengue, Chikungunya and Zika Transmission

**DOI:** 10.1371/journal.pone.0170079

**Published:** 2017-02-15

**Authors:** Catherine A. Pruszynski, Lawrence J. Hribar, Robert Mickle, Andrea L. Leal

**Affiliations:** 1 Florida Keys Mosquito Control District, College Road, Key West, Florida, United States of America; 2 REMSpC Spray Consulting, Welsh Drive, Ayr, ON, Canada; Universidade Federal do Rio de Janeiro, BRAZIL

## Abstract

**Background:**

*Aedes aegypti* is a container-inhabiting mosquito and a vector of dengue, chikungunya, and Zika viruses. In 2009 several cases of autochthonous dengue transmission were reported in Key West, Florida, USA prompting a comprehensive response to control *A*. *aegypti*. In Key West, larvae of this mosquito develop in containers around human habitations which can be numerous and labor intensive to find and treat. Aerial applications of larvicide covering large areas in a short time can be an efficient and economical method to control *A*. *aegypti*. *Bacillus thuringiensis israelensis* (*Bti*) is a bacterial larvicide which is highly target specific and appropriate for wide area spraying over urban areas, but to date, there are no studies that evaluate aerial spraying of *Bti* to control container mosquitoes like *A*. *aegypti*.

**Methodology:**

This paper examines the effectiveness of aerial larvicide applications using VectoBac^®^ WG, a commercially available *Bti* formulation, for *A*. *aegypti* control in an urban setting in the USA. Droplet characteristics and spray drop deposition were evaluated in Key West, Florida, USA. The mortality of *A*. *aegypti* in containers placed under canopy in an urban environment was also evaluated. Efficacy of multiple larvicide applications on adult female *A*. *aegypti* population reduction was compared between an untreated control and treatment site.

**Conclusions:**

Droplet characteristics showed that small droplets can penetrate through dense canopy to reach small containers. VectoBac WG droplets reached small containers under heavy canopy in sufficient amounts to cause > 55% mortality on all application days and >90% mortality on 3 of 5 application days while controls had <5% mortality. Aerial applications of VectoBac WG caused significant decrease in adult female populations throughout the summer and during the 38^th^ week (last application) the difference in adult female numbers between untreated and treated sites was >50%. Aerial larvicide applications using VectoBac WG can cover wide areas in a short period of time and can be effective in controlling *A*. *aegypti* and reducing *A*. *aegypti*-borne transmission in urban areas similar to Key West, Florida, USA.

## Introduction

Domestic container mosquitoes have adapted to colonize artificial containers produced by humans such as tires, cemetery vases and many different types of discarded trash or debris that can hold a small amount of water [1,2]. This characteristic is most likely a carryover from their sylvan form whose oviposition sites are tree holes and bamboo nodes that collect less than 1L of rainwater [1]. Container mosquitoes are versatile in habitat and choice and although they are predominantly found inhabiting small containers in Florida, in some parts of the world they can also develop in larger containers such as earthen jars and cisterns [3][4]. Container mosquitoes are vectors for many diseases, and controlling their population is an important task for public health agencies. The traditional approaches to larval mosquito control are to find the larval source and treat it with appropriate chemical or destroy the source, but container mosquito larvae can develop in such minimal amounts of water that finding these larval sources becomes very labor intensive especially in countries like the USA[5].

*Aedes aegypti* is one such important container mosquito species established in tropical and sub-tropical locations including the southern United States [6]. It is one of the principal vectors of dengue, chikungunya, and Zika, and has caused epidemics of all three diseases throughout the world [7–9]. In Key West, *A*. *aegypti* can be found associated with human habitats as larvae occupying small water-holding containers and as adults frequently feeding on humans [2,10]. The ubiquitous presence of small containers close to human habitations in urban Key West combined with the anthropophilic nature of *A*. *aegypti* makes these vectors efficient in transmitting mosquito-borne viruses. Most houses in Key West are air-conditioned and *A*. *aegypti*-positive containers are predominantly located outside of the houses.

Recent estimates indicate that there are 390 million dengue infections per year of which 96 million are considered clinical infections [11]. Chikungunya caused explosive outbreaks throughout the western hemisphere in 2014. The World Health Organization (WHO) estimated that over 1.3 million cases of chikungunya occurred in Central and South America and the Caribbean by April 2015 [12]. Newly emerging Zika virus presents as a mild flu-like illness in adults, but recent epidemiological studies have indicated a possible link between Zika infections in pregnant women and an increase in microcephaly in newborns. In early 2016, the WHO declared Zika a Public Health Emergency of International Concern, and cases of the virus are spreading to new locations [13].

Mosquito-borne viral epidemics are on the rise and globalization has reduced the geographical barriers for disease movement. Cases of travel-associated dengue, chikungunya, and now Zika are reported every year in non-endemic areas. Viral infections such as chikungunya and Zika had not been previously reported in the new world, but current outbreaks are linked to travel-associated transmission from Africa and Asia. As of 7 December 2016, there have been 4,389 travel-associated cases of Zika reported in the continental United States and 185 autochthonous cases [14]. The year-round presence of *A*. *aegypti* is conducive to a potential outbreak of human disease if the virus is introduced under the suitable ecological conditions. As there is no widely available vaccine or cure for dengue, chikungunya, nor Zika, vector control remains the only means to reduce transmission of disease.

The Florida Keys Mosquito Control District (FKMCD) was established in 1951 in response to the nuisance and public health impact mosquitoes were having on the growing population of residents of the Florida Keys. Although there are more than 40 mosquito species found within the area, *A*. *aegypti*, *A*. *taeniorhynchus* and *Culex quinquefasciatus* are the principal species that are targeted by the FKMCD due to their disease-causing potential and annoyance. Imported dengue cases have been reported in Key West, but the last known autochthonous transmission of dengue in Florida had been in 1934 [15].

However in 2009, an autochthonous outbreak of dengue fever transmitted by *A*. *aegypti* was reported from Key West, Florida [15,16]. This outbreak resulted in 22 reported cases of dengue [17]. In January 2010, the district hired additional domestic inspectors to search homes for *A*. *aegypti* with five inspectors covering more than 6,000 residences and 400 known cisterns. This resulted in properties being visited once every 30 days. The primary methods of abatement included emptying water-holding containers and hand-treating with S-Methoprene, Spinosad, and *Bacillus thuringiensis israelensis* (*Bti*) granules. The district also implemented a rigorous public outreach program that extended to door knockers, radio ads, and a weekly 30-minute television program. Nightly truck-mounted ULV spraying also occurred in areas with positive human cases as an added safeguard, although effectiveness of this practice against a diurnal species is debatable. Despite these control efforts, mosquito populations steadily increased through the summer months and 65 dengue cases were reported in Key West by the end of 2010 [18]. Targeting the *A*. *aegypti* larvae in this urban area by aerial spray of bacterial larvicide was explored to control the outbreak.

Treatment with *Bti* is an important method of mosquito abatement for many control operations. Proteins expressed by this natural, and environmentally benign, bacterium cause mortality upon ingestion specifically to “lower flies” of the Dipteran sub-order Nematocera. The families Culicidae and Simulidae are highly susceptible, whereas other Nematocera are orders of magnitude lower in susceptibility [19]. This active ingredient is ideal for ephemeral water-holding areas because it causes mosquito larval mortality within 24–48 hours and does not affect non-target species [20,21]. Bacterial larvicides have been thoroughly reviewed for safety to non-target organisms including mammals, and are recommended for application in drinking water storage containers by the WHO [22,23]. Numerous studies have shown that *Bti* larvicides are target specific and cause no harm to other organisms including humans [24–27]. Hence aerial applications using small droplets of *Bti* are ideal choices for area wide aerial spraying in heavily populated urban area like Key West.

VectoBac^®^ WG (Valent Biosciences Corporation, Illinois, USA) is a water dispersible granule formulation of *Bti* strain AM65-52. This product has been reviewed by the WHO Pesticide Evaluation Scheme and is approved for organic applications [28]. Spray application of VectoBac WG with equipment designed to generate small drops containing suspended *Bti* strain AM65-52 offers efficiency in reaching the numerous difficult to find and treat habitats of container mosquitoes [5]. Ground spray application of VectoBac WG for the control of container mosquitoes has been evaluated in numerous studies since 2004 [29–31]. Applications using backpack sprayers and thermal foggers at rates of ranging from 400 to 800 grams per hectare have resulted in 96–100% control of *A*. *aegypti* larvae in small containers within the treated areas [32–34]. Truck mounted applications delivered 87–100% control at similar rates [32]. Residual control of immature *A*. *aegypti* in cups was also reported up to 9 weeks following spray application of VectoBac WG [32,34]. Reductions in adult container *Aedes* spp. densities following repeated spray applications of the product using backpack and truck spraying have also been demonstrated, with percent reductions in ovitrap indices ranging from 56 to 87% [32,35]. Economic analysis have indicated that locating and treating container mosquito habitats using manual labor is prohibitively expensive especially in countries similar to the USA [5,34]. Although door-to-door campaigns to empty containers can also serve as an education and awareness program, targeting small containers with VectoBac WG droplets is less expensive and similarly effective than physically finding and emptying the containers in Key West [5]. The Florida Keys Mosquito Control District has a strong public education program that strives to encourage residents to dump out standing water around their homes and businesses.

Past studies have been conducted only with ground spraying equipment which is effective but labor intensive when large areas are needed to be covered in case of an epidemic. Aerial applications of VectoBac WG can cover large areas in a short time which can offer a significant benefit especially during vector-borne disease epidemics. Previous studies using aerial applications of *Bti* have been done using ice crystals or *Bti* granules in flood water habitats away from human dwellings [36]. Aerial larvicide applications using small droplets measuring approximately 150 microns need to be conducted at an approximate altitude of 30m or higher from the ground. Small larvicide droplets released at such altitudes in residential urban areas like Key West need to reach containers breeding *A*. *aegypti* larvae under dense vegetation or debris cover in sufficient amounts to cause high larval mortality. While the droplets need to be small enough to penetrate vegetation it also needs to be large enough to come down to the ground to deposit in small containers with sufficient amounts of *Bti* to be lethal to *A*. *aegypti* mosquitoes developing in those containers. There have been no previous studies on the feasibility of such wide liquid area aerial larviciding using small droplets for container mosquitoes such as *A*. *aegypti* in urban areas similar to Key West.

The objective of this study was to evaluate: 1) deposit pattern, downwind deposition and efficacy of VectoBac WG droplets produced with aerial spray equipment; 2) whether VectoBac WG droplets can penetrate and reach small containers under canopy in an urban setting during operational aerial spraying; and 3) whether repeated VectoBac WG aerial spray applications in Key West, Florida, USA are effective in reducing the adult female populations of *A*. *aegypti*.

## Methods

### Ethics Statement

All aerial spraying and mosquito control activities were conducted in compliance with Florida State Statute 388 and rule chapter 5E-13 of the Florida Administrative code allowing the power to perform work on both public and private lands. All surveys and collections were made by county mosquito control professionals. Private yards and other private lands were entered only after oral and/or written consent was obtained from the individual owners/residents. No specific permits were required for the collection of mosquitoes and these studies did not involve endangered or protected species. Ethical Committee approval was not required for this study.

### 1) Deposit Pattern and Swath Analysis

Two characterization tests were completed with permission on Boot Key, a private undeveloped island, on March 14th, 2012. For each test, a single pass at 30 m altitude was made using 6-AU5000 (Micron Group, Herefordshire, UK) atomizers mounted on a Bell 206L helicopter (Bell, Texas, USA) flying at 130 km/hh. The application rate was set at 0.56kg VectoBac WG applied in 4.7 L of water per hectare. Assuming a 60 m swath, flow rate was set to 10.2 L/min through each of the 6-AU5000 atomizers coupled with a fan blade setting of 40 degrees which provided rotational speeds between 4000–5000 rpm. The spray mix contained 12% VectoBac WG by weight and was dyed red with 2% granular red dye (Allura Red- FD&C Red #40, Sensient Chemicals, USA).

Just upwind of the flight and sample line intersection, a 6 m tower was erected to log one-minute average wind speed and direction during the trials. Sample sites were established along a 457 m section of abandoned road, perpendicular and to the west of the N-S flight line. At 15 m intervals, 3 sets of 2 types of sampling devices were placed: 1) Kromekote card (Mohawk Fine Paper, New York, USA) measuring 7 x 11 cm for ground deposit and 2) empty clear polystyrene sample jars (Mold-Rite Plastics, New York, USA) measuring 6.4 cm in diameter by 6.4 cm deep for post-spray bioassay. At each downwind distance, sample sites were placed along the mid-line of the roadway, under a moderate canopy in the tree line to the south of the road and under a heavy mangrove canopy to the north of the road. Each site was photographed to assess obscuration from intervening foliage that drops could encounter before depositing in/on the samplers. Obscuration was measured by an in-house program that analyses the picture using a range of luminosity values and assesses the fraction (number of pixels) of the picture that lies above this value (fraction of picture that is sky). As this luminosity value is varied the fraction stabilizes at a critical luminosity and the fraction at that point is used to calculate the obscuration value (i.e. 1—the fraction of sky)*100%.

The first test with a standard atomizer fan blade setting of 40 degrees was completed at 08:45, approximately one hour after sunrise. Winds on site at 6 m were variable at 6–18 km/h with an average direction near 45 degrees consistent with forecast winds. However, instantaneous wind direction up to and during the spray was variable, ranging from near parallel to the flight line to 60 degrees. Sample collection starting at the flight line end of the sample line commenced about 10 min after the aircraft had passed. The second characterization test using a fan blade setting of 35 degrees was completed at 09:45, one hour later. However, the thermals changed and the day became hotter by the time the second characterization was completed hence the data only from the first characterization was analyzed and reported here.

After application, the Kromekote cards that were placed on the ground were brought to the laboratory and scanned at 1200 dpi using a canon LiDE 100 scanner (Canon, Tokyo, Japan). The scanned pictures were then processed through Stainalysis software, a freeware program available at www.remspc.com. The droplets of spray mixture produce stains on the Kromekote cards. The software converts the stain into droplet diameter based on the spread factor of the droplets. The spread factor is a relationship between the stain size to drop size [37]. Spread factor is a function of formulation and to a lesser degree humidity conditions. The stain analysis software measures the droplet sizes in volume mean diameter (VMD) and volume of droplets in each scanned picture.

After application, the vials used for bioassay evaluations were capped and brought to the laboratory. The vials were filled with 100ml of filtered tap water and 5 *A*. *aegypti* 2nd instar laboratory-reared larvae were introduced. The cups were monitored 2 hours, 4 hours, and 24 hours after addition of larvae to determine the larval mortality.

### 2) Droplet Penetration

**Fig 1:**
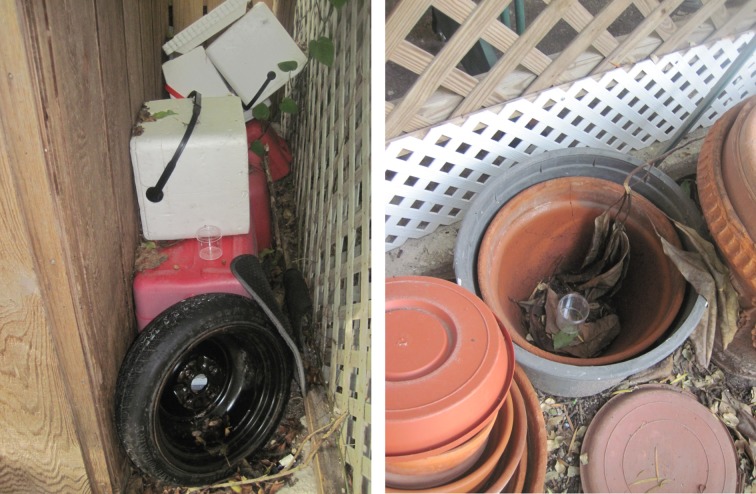
Picture of bioassay container placed under heavy urban canopy in Key West, Florida.

Aerial applications of VectoBac WG were conducted in the Old Town area of Key West in 2011. Applications were made on 01/21/2011, 01/29/2011, 02/19/2011, 02/26/2011, 03/15/2011, 04/9/2011, 05/21/2011, and 06/4/2011. All applications were conducted in the early morning time between 06:00 and 08:00. Before application polystyrene cups were randomly placed under heavy canopy in the application area in private residents with owner permission. The canopy here refers to both heavy vegetation and buildings. (Fig 1). Thirty-one cups were used for each trial. During the time of application 10 control cups were carried to the field where the applications were made but were covered in order to prevent the application droplets from reaching them. After application the treatment and control cups were brought back to the laboratory and filled with 100ml water and 5 *A*. *aegypti* 2nd instar laboratory-reared larvae and 5 *C*. *quinquefasciatus* 2nd instar laboratory-reared larvae. Although the original aim was to test the efficacy of aerial applications of VectoBac WG against *A*. *aegypti* larvae, bioassay was also conducted with *C*. *quinquefasciatus* for operational evaluation as it is the other most common urban dwelling mosquito within the Key West area. The cups were monitored and mortality was recorded at 24 hrs post collection. The mortality between control and treatment on each date was compared with a t-test.

### 3) Aerial Application of VectoBac WG to Control *A*. *aegypti*

A treatment site and untreated control site of approximately 405 hectares each were established for evaluating the efficacy of VectoBac WG aerial application in 2013. The treatment site was an area of Old Town Key West and the untreated control site was an area two miles northeast of the spray zone. Treated and untreated control sites received the same mosquito control attention that included house-to-house mosquito inspection, dumping of standing water, and treatment of large containers with (S)-methoprene (Altosid^®^, Central Life Sciences, Illinois, USA) and spinosid (Natular®, Clarke, Illinois, USA). Since the untreated control site is also a residential area with heavy *A*. *aegypti* infestation during the peak season some level of *A*. *aegypti* intervention methods (described above) had to be conducted. The only difference between the sites is that the untreated control area received no aerial treatment with VectoBac WG whereas the treatment area received the aerial larvicide treatment.

Seven BG-Sentinel™ traps (Biogents, Regensberg, Germany) were set in each treatment and control areas in private residences with owner permission, for a total of 14 traps. Traps were set once a week, and were baited with carbon dioxide (CO_2_) as condensed dry ice pellets and a BG lure which is designed to attract anthropophilic mosquitoes by mimicking scents found on human skin [38]. Although male mosquitoes are also attracted to the BG Sentinel, female mosquitoes are the principal target of these traps. Traps were left in the field for 19 hours. Trapping started on January 1, 2013 and continued until November 8, 2013. Trap net contents were stored in a freezer, and later mosquitoes were identified and counted.

Ten aerial VectoBac WG applications were conducted during the rainy season between May through September within the test area in Key West, FL. There were five weekly applications starting on May 31, 2013 (05/31/13, 06/7/2013, 06/14/2013, 06/21/2013, 06/28/2013) followed by four bi-weekly applications (07/12/2013, 07/26/2013, 08/9/2013, 08/23/2013), and one application that was delayed due to weather on September 19, 2013. Data from previous years have indicated that an increase in *A*. *aegypti* population density coincides with rainfall accumulation during the summer months [2]. By repeating weekly missions in the beginning of the rainy season followed with bi-weekly missions through July, the rational was that the initial population would be suppressed at the start of the rainy season and maintained in low numbers through the entire summer. The aerial applications were optimized with the droplet characterization study conducted earlier (See Results). The Bell 206L helicopter was used with six AU5000 Atomizers and a 60 m lane separation (swath width) at an altitude of 30 m with a pitch of 40° flying at 130 km/h.

The female *A*. *aegypti* BGS trap counts before (before 22^nd^ week) and after the aerial application were compared between control and treatment sites using a negative binomial regression with a log link function which is recommended analysis for overdispersed count data [39][40]. The control and treatment site female *A*. *aegypti* weekly numbers were fit with a locally weighted polynomial regression (LOESS model) in order to visualize the data [41]. The plot was made using GGPLOT2 (http://ggplot2.org/) package and both the plot and statistical analysis were conducted on the open source software platform R studio (www.rstudio.com) [42]

## Results

### 1) Deposit Pattern and Swath Analysis

**Fig 2:**
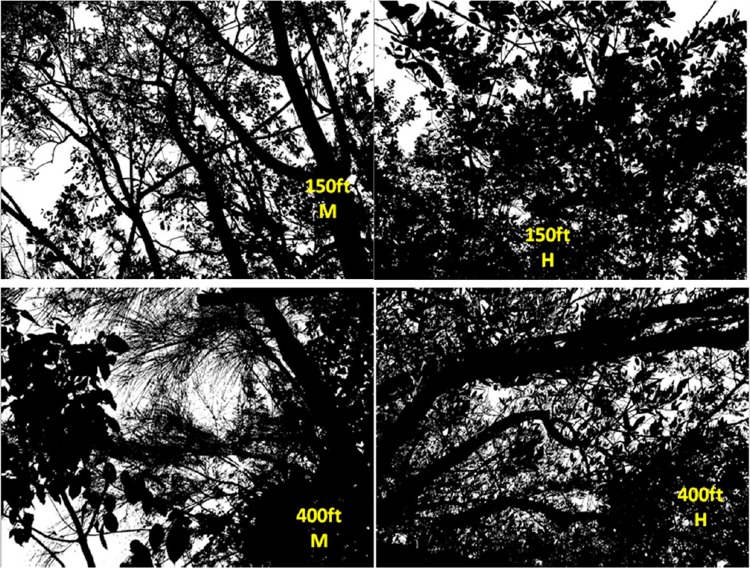
Photographs of the two canopy types used for droplet characterization.

**Fig 3:**
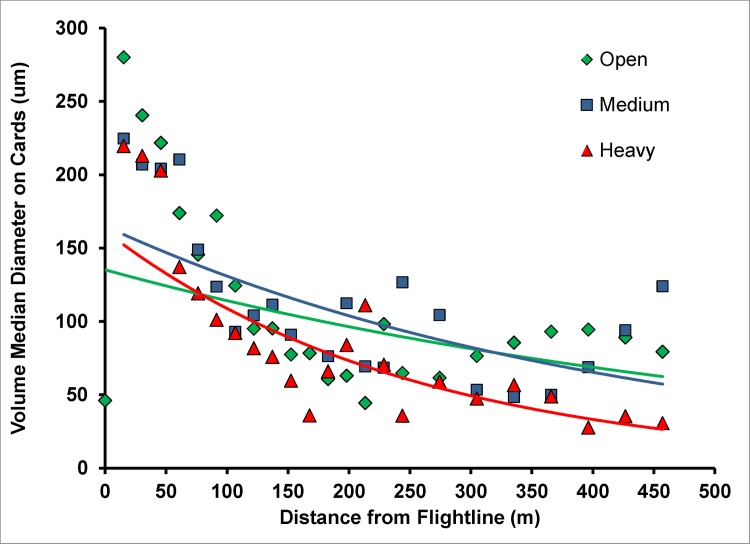
VMD of the droplets that deposited on the Kromekote cards at different distances from the flight line in the open (green inverted squares, green line), medium canopy (blue squares, blue line) and heavy canopy (red triangles, red line).

Two canopy covers, Medium and Heavy were differentiated based on photos taken upward through the canopy at each sample site. Obscuration was calculated as the percent of photo not showing sky. Generally, obscuration at the medium sites averaged 69% and at the Heavy canopy sites 86% (Fig 2). Deposit was detected out to 457 m downwind of the spray line. Analyses of the ground cards showed a rapid decrease in VMD over the first 214 m of the sample line (Fig 3) with large drops depositing close to the flight line. At distances near 152 m, volume median diameters were less than those near the flight line. Beyond 214 m, VMD was relatively constant, remaining less than 100μm, indicative of small drops having drifted to the far field. At distances less than 152 m where the majority of the spray deposited, drops on cards placed under the medium and high-density canopies were somewhat smaller as the larger drops deposited onto foliage.

**Fig 4:**
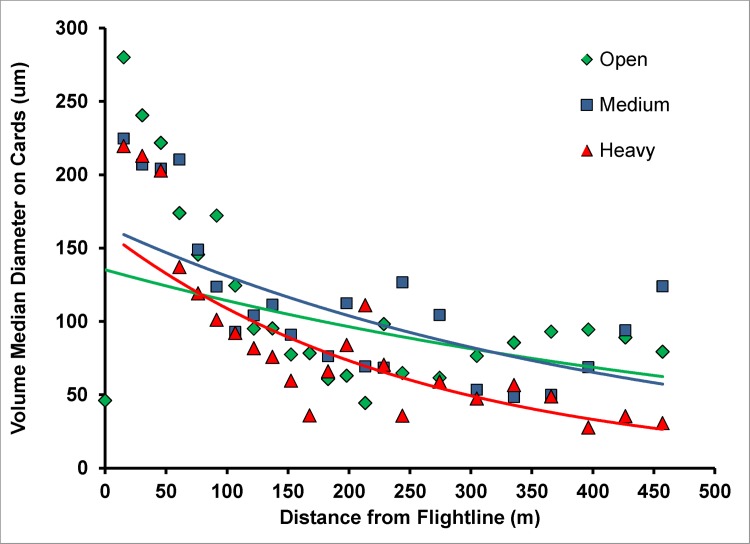
Droplet density on cards (number of droplet/cm^2^) at different distances from the flight line in the open (green inverted squares, green line), medium canopy (blue squares, blue line) and heavy canopy (red triangles, red line).

Droplet density (drops/cm^2^) produced a significantly different pattern and was much broader. The majority of drops found on cards were less than 100μm. Due to their small size, drift over extended distances resulted in significant deposit of drops <100 μm in the far field although their overall associated volume was small. In the open, drop density decreased by a factor of ten over the 457 m sample line (Fig 4). Canopy decreased the number of drops reaching the ground, with the reduction in droplets within the first 150 m due to the foliage density in the canopy.

**Fig 5:**
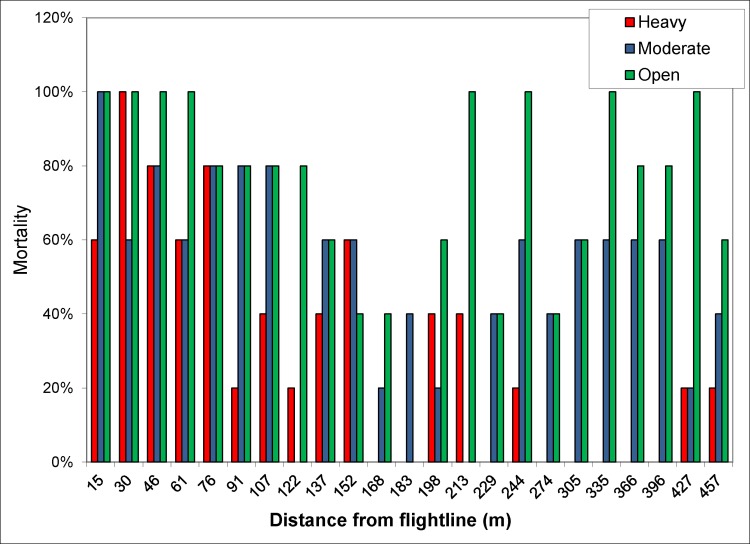
Mortality of *A*. *aegypti* larvae at 2 hours after the bioassay containers were filled with water and larvae were added in the droplet characterization study. These containers were placed at fixed distances away from the flight line. Containers placed in open areas are in green, moderate (medium) canopy are in blue, heavy canopy is in red.

Bioassays showed rapid mortality (2 hours) in containers exposed in the first 75 m downwind of the spray line (Fig 5). The larval mortality was <5% in the control cups and were not included in the graph. The larval mortality at 4hrs showed consistent mortality regardless of obscuration (Canopy cover). The droplet deposit comparison with larval mortality also indicated that the amount of product deposit required for larval mortality in the cups under canopy was <5% application rate. The high mortality even at low deposit rates could be attributed to evaporation during drift resulting in more concentrated droplets being deposited at ground. Overall the droplet analysis indicated that 60–75 m would be an effective swath width for aerial application of VectoBac WG with the application system tested

### 2) Droplet Penetration

**Fig 6:**
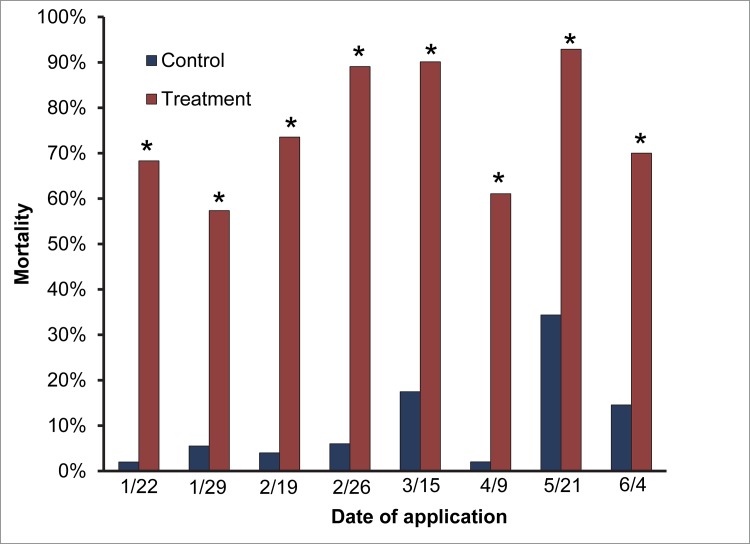
Larval mortality of *A*. *aegypti* in the bioassay containers placed under the buildings/canopy during the operational aerial larvicide spraying of VectoBac WG in Key West. Bars with stars indicate that they were significantly different than the untreated control bioassay cups.

Since there was no difference in mortality between *A*. *aegypti* and *C*. *quinquefasciatus*, the data were combined and analyzed as total larval mortality. Larval mortality was significantly different for control and treatment cups at all application rates (P = <0.0001, Fig 6). Mortality in the treatment cups stayed above 55% on all the days when applications were made indicating that the droplets of VectoBac WG reached hidden containers under heavy canopy. Mortality was 90% in the cups for 3 days out of the 5 days when applications were made and when control mortality was below 5%. The control mortality stayed below 20% for all days except one day when it reached 30%.

### 3) Aerial Application of VectoBac WG to Control *A*. *aegypti*

**Fig 7:**
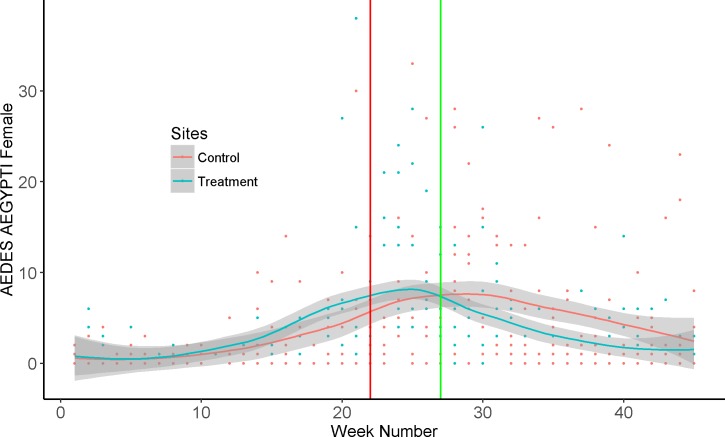
Locally weighted polynomial regression (LOESS model) curves plotted for the weekly counts of *A*. *aegypti* female numbers. The vertical red line indicates the beginning of weekly *Bti* applications and the green line indicates bi-weekly applications.

Sampling with BG Sentinel traps from Jan to Oct constituted 45 weeks of data. The number of female *A*. *aegypti* remained close to zero for the first 10 weeks of pre-treatment sampling. After the 10th pre-treatment week, the female *A*. *aegypti* number from the treatment site increased at a higher rate than control site (Fig 7). The weekly aerial applications of VectoBac WG were started on the 22nd week and the biweekly applications were started on the 28th week (Fig 7). The negative binomial regression indicated no significant difference (P = 0.7211) between the control and treatment *A*. *aegypti* female numbers before *Bti* applications (22^nd^ week) were started. But there was a significant difference in *A*. *aegypti* female numbers (P = <0.0002) between the two sites after *Bti* applications were started. The coefficient estimates indicated that after the *Bti* applications were started, for every unit increase in control site *A*. *aegypti* female numbers there was a 0.67 unit decrease in treatment site numbers (Fig 7) Populations of *A*. *aegypti* from the control site showed general trend of reduction from the 30th week indicating the end of the peak season but this reduction in population in the treatment site was steeper than the control site. The last biweekly aerial application was conducted on the 38th week and *A*. *aegypti* totals from the treatment site stayed lower than the control site beyond the 40th week of sampling (Fig 7). The adult *A*. *aegypti* female populations were >50% lower in the treated site compared to the untreated control site at the 38^th^ week.

## Discussion

The wet season in Key West, FL begins in the month of May and surveillance data show that *A*. *aegypti* numbers increase and attain their peak during the month of June [39]. The results showed that larvicide droplets from an aerial application can reach small container habitats under heavy canopy to cause significant mortality to *A*. *aegypti* larvae. The reduction in numbers was achieved despite a bigger spike in *A*. *aegypti* population in the treatment areas compared to the untreated control area (. 7).

*Aedes aegypti* is the predominant container mosquito in the Florida Keys, and utilizes a wide variety of container habitats including tires, buckets, discarded plastic containers, flower pots, plant trivets, garbage cans, and bromeliads for larval development. In both wet and dry season larval surveys, about half of all containers examined contained water. House, container, and Breteau indices all increased during the wet season [2]. The strategy to use weekly applications of VectoBac WG during the peak wet season months of May and June followed by bi-weekly applications during July and August served as an effective strategy in reducing the populations of *A*. *aegypti* by >50% in the treatment area compared to the untreated control area.

Messenger et al. [43] conducted a dengue seroprevalence study in March 2012 to test the presence of dengue antibodies among the permanent (residing 1 year or more) Key West residents. They found that 12% of the participants tested positive for dengue antibodies and had travel history to dengue endemic areas in the 2 years preceding the test year. They also found that 3.5% of the participants tested positive but had no travel history to dengue endemic areas indicating that they acquired the infection locally. Most likely these participants carried the infection, but were never tested for dengue. The study indicates the presence of a high number of travel-associated cases and suggests possible locally-acquired cases of dengue within Key West. The Key West Chamber of Commerce reports that more than 2.6 million people visit the area every year [44]. Reductions in autochthonous cases of *A*. *aegypti*-vectored disease in Key West could be attributed to the use of air conditioning and window screens that minimize human contact with the vector, but the authors would like to argue based on the data presented here that robust vector control measures including aerial larviciding in Key West are effective strategies that suppress *A*. *aegypti* populations.

Bacterial larvicides are target specific and generally do not offer long term control, and in this experiment, we found weekly and biweekly application suitable for long-term suppression. However, previously published laboratory and field experiments showed some residual control with VectoBac WG. Setha et al. [45] reported that VectoBac WG provided 12 weeks of *A*. *aegypti* control when applied directly to drinking water containers in Cambodia. Williams et al. [32] reported that when VectoBac WG applied with truck mounted foggers provided 6 weeks control in the laboratory. The droplet characteristics for an aerial application of VectoBac WG, especially evaporation and other environmental factors will be different from backpack and truck mounted fogging applications. Further studies are needed to evaluate whether aerial applications of VectoBac WG will provide any residual control of container mosquitoes. The results described here add to the tools that could be used to suppress these vectors. Similarly there are other tools using auto-dissemination of insect growth regulators to control these mosquitoes. In these auto-dissemination studies the skip oviposition behavior of female *A*. *aegypti* is used to disseminate the growth regulator to the larval habitats [46][47]. Growing resistance to pesticides is a major problem in effectively controlling *A*. *aegypti*. Among larvicides that are commonly used *A*. *aegypti* are resistant to temephos in many part of the globe [48,49]. They are also resistant to different types of pyrethroids [50] and organophosphates [51]. In California, USA the *A*. *aegypti* populations are resistant to some pyrethroids except the Class II pyrethroid, deltamethrin [52]. There have been no reports on *A*. *aegypti* resistance to *Bti* but there are studies that show that *A*. *aegypti* resistant to pyrethroids and organophosphates are still susceptible to *Bti* [48] and hence *Bti* can be an effective tool for resistance management.

Domestic *A*. *aegypti* are found in and around homes as adult females feeding on humans and as larvae predominantly occupying small water-holding containers including plastic containers and trash in the USA. In some parts of the world *A*. *aegypti* larvae can be found in containers inside human dwellings demonstrating the versatility of this species [53]. These characteristics have enabled *A*. *aegypti* to become the most important vector of many viruses including dengue, chikungunya, and Zika. As there is limited availability for the dengue vaccine and no vaccines or prescribed treatments for chikungunya or Zika, control of the vector remains the most important factor for abating virus transmission. The aerial application of a liquid bacterial larvicide can be an effective strategy to control *A*. *aegypti* in large urban areas similar to Key West in a short time, and may help to prevent outbreaks of *A*. *aegypti*-vectored disease. If the predominant *A*. *aegypti* habitats in a given area are large and or inaccessible to aerial droplets then aerial application may not be an effective method in these areas. But if the predominant *A*. *aegypti* habitats are small containers within the reach of aerial droplets like those found in Key West, FL then aerial application of liquid *Bti* larvicide may be an effective strategy. The aerial methods described here could be easily replicated in areas similar to Key West with most aircraft that are being used for agricultural applications. Larval habitats of *A*. *aegypti* vary across the globe from large earthen jars used for drinking water storage in Indonesia [3] to small containers such as small discarded containers in Puerto Rico [54]. Any strategy developed for a particular region depends on the local habitat characteristics of *A*. *aegypti* and the infrastructure available in the region. The study described here shows that wide area applications using small droplets of *Bti* (AM65-52) can be used effectively to penetrate canopy and other obstructions to reach the majority of small containers, and will cause significant mortality to larvae in said containers. The authors sincerely hope that the methods and results described here are helpful in developing successful strategies to control *A*. *aegypti*, a vector responsible for causing significant impact on human health across the globe.
